# Ferritin heavy chain (FTH1) exerts significant antigrowth effects in breast cancer cells by inhibiting the expression of c‐MYC

**DOI:** 10.1002/2211-5463.13303

**Published:** 2021-10-06

**Authors:** Amjad Ali, Jasmin Shafarin, Rola Abu Jabal, Nour Aljabi, Mohamad Hamad, Jibran Sualeh Muhammad, Hema Unnikannan, Mawieh Hamad

**Affiliations:** ^1^ Research Institute for Medical and Health Sciences University of Sharjah United Arab Emirates; ^2^ Department of Basic Medical Sciences College of Medicine University of Sharjah United Arab Emirates; ^3^ Department of Medical Laboratory Sciences College of Health Sciences University of Sharjah United Arab Emirates

**Keywords:** breast cancer, c‐MYC, FTH1, G9a, iron metabolism

## Abstract

Overexpression of ferritin heavy chain (*FTH1*) often associates with good prognosis in breast cancer (BCa), particularly in the triple‐negative subtype (triple‐negative breast cancer). However, the mechanism by which *FTH1* exerts its possible tumor suppressor effects in BCa is not known. Here, we examined the bearing of *FTH1* silencing or overexpression on several aspects of BCa cell growth *in vitro*. *FTH1* silencing promoted cell growth and mammosphere formation, increased *c‐MYC* expression, and reduced cell sensitivity to chemotherapy. In contrast, *FTH1* overexpression inhibited cell growth, decreased c‐MYC expression, and sensitized cancer cells to chemotherapy; silencing of *c‐MYC* recapitulated the effects of *FTH1* overexpression. These findings show for the first time that *FTH1* suppresses tumor growth by inhibiting the expression of key oncogenes, such as *c‐MYC*.

## Graphical abstract

Similar to chemotherapy and iron chelation, increased expression of ferritin heavy chain 1 (FTH1) suppresses the expression of cellular myelocytomatosis and G9a in breast cancer (BCa) cells. This is associated with increased cellular iron influx, storage, and release and reduced BCa cell growth. These findings suggest that FTH1 acts as a tumor suppressor in BCa.

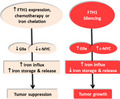

**Abbreviations**
5‐FU: fluorouracil
BCa: breast cancer
BET: bromodomain and extraterminal
BRD: bromodomain
c‐Myc: cellular myelocytomatosis
DFO: deferoxamine (desferal)
FPN: ferroportin
FT: ferritin
FTH1: ferritin heavy chain
FTL: ferritin light chain
G9a (EHMT2): euchromatic histone lysine methyltransferase 2
HER2: human epidermal growth factor receptor 2
IFNγ: interferon gamma
IRG: iron regulatory gene
IRP‐2: iron regulatory protein 2
SAHA: suberanilohydroxamic acid
siRNA: small interfering RNA
STEAP3: six‐transmembrane epithelial antigen of the prostate 3
TCGA: the cancer genome atlas
TFR1: transferrin receptor 1
TNBC: triple‐negative breast cancer

Breast cancer (BCa) is the most common malignancy in women and is one of the three most common cancers worldwide [1]. A variety of therapeutic options are available including surgery, radiation, chemotherapy, and hormone therapy. However, to determine the optimal treatment course, in addition to evaluation of the histologic subtype, several clinical and pathological characteristics are evaluated. Due to these challenges, BCa remains a clinical enigma in terms of prognostic evaluation and therapeutic options [2]. Disruption of iron metabolism is known to be a two‐edged sword in modulating carcinogenesis [3]. Hence, understanding the role of key iron metabolism‐related proteins in the development and progression of BCa may offer an opportunity to address these challenges.

Ferritin (FT) is a protein complex that stores iron in a bioavailable, nontoxic form [4]. The bulky 24‐subunit cage‐like structure consists of a variable number of repeats of the FT heavy chains (FTH1) and the FT light chain (FTL) subunits [5]. Although mammalian FTH1 and FTL proteins share significant sequence homology, they are functionally distinct [6]. FTH1 exhibits significant ferroxidase activity resulting from glutamic acid residues that serve as metal ligands [7] and help in rapid iron uptake [8, 9]. FTL on the other hand has multiple carboxy groups that line the shell cavity to aid in iron nucleation [9]. Increased levels of labile iron in the cytoplasm trigger the iron regulatory protein (IRP‐2), acting on iron‐responsive elements at the 3′UTR of FT mRNA to increase FT synthesis and enhance iron storage [10].

Previous work has shown that FTH1 expression is increased several human cancers including hepatocellular carcinoma [11], Hodgkin's lymphoma [12], and pancreatic cancer [13]. This suggests that FTH1 may exert oncogenic effects possibly by enhancing angiogenesis [14, 15, 16], inhibiting apoptosis [17, 18, 19, 20], and/or inducing epithelial–mesenchymal transition [21, 22]. Other studies have shown that increased FTH1 expression induces tumor‐suppressive effects in cancer by enhancing apoptosis [23, 24, 25, 26] and/or directly interacting with and activating p53 [27]. Concerning BCa, while several previous studies have suggested that increased FTH1 expression associates with increased resistance to chemotherapy [17, 18, 19], others have shown that FTH1 can serve as a significant tumor suppressor in BCa. FTH1 was previously reported to be reduced in BCa cells [28, 29, 30] and that transformation and progression of epithelial BCa tumors often associate with decreased FTH1 and ferroportin (FPN) expression and increased transferrin receptor 1 (TFR1) expression [31, 32]. Increased FTH1 expression is now considered as a favorable prognostic marker in triple‐negative BCa (TNBC) [33, 34]. Additionally, BCa tissues with high levels of FTH1 tend to be highly enriched for interferon gamma‐producing CD8^+^, but not CD4^+^, T cells, suggesting that FTH1 modulates the adaptive immune response within the tumor microenvironment [33].

These observations notwithstanding, the exact role of FTH1 in BCa molecular subtypes remains ambiguous. Additionally, the question of whether FTH1, as part of the iron‐storing protein FT, can interact with key oncogenes that alter cellular iron availability such as cellular myelocytomatosis (c‐MYC) and G9a and how such interactions influence BCa cell growth has yet to be addressed. To this end, data mining approaches were used to examine the expression/coexpression patterns of FTH1, c‐MYC, bromodomain (BRD), and G9a genes in different BCa molecular subtypes. Additionally, cell growth, migration, and mammosphere formation along with the expression status of oncogenes that are directly or indirectly related to cellular iron metabolism were evaluated in MCF‐7, MDA‐MB‐231, SKBR3, and JIMT1 cells following small interfering RNA (siRNA)‐mediated knockdown of FTH1, c‐MYC, and/or G9a in the presence/absence of various anticancer agents, vitamin C, or the iron chelator deferoxamine (DFO). Our data show that FTH1 and c‐MYC can reciprocally regulate the expression of one another and that this interplay has a significant bearing on the extent of BCa cell growth and migration.

## Materials and methods

### Cell culture

Human BCa cell lines were as follows: MCF‐7 (RRID CVCL_0031), MDA‐MB‐231 (RRID: CVCL_0062), and SKBR3 (RRID: CVCL_0033; CLS Cell Lines Service GmbH, Eppelheim, Germany). These cell lines were mycoplasma‐free and were authenticated using short tandem repeat (STR) genotyping within the last 3 years and were verified to be identical with the STR profile in reference databases (CLS Cell Lines Service GmbH). The JIMT1 cell line, which was purchased from AddexBio (San Diego, CA, USA; Catalog #: C0006005), was also used in this study. The choice to use these cell lines was based on the fact that MCF‐7 is representative of Luminal A subtype, MDA‐MB‐231 of TNBC subtype, and SKBR3 and JIMT1 of HER2^+^ subtype [35, 36]. Cells were cultured in Dulbecco's Modified Eagle Medium (DMEM) with high glucose (D 6429) supplemented with 10% FBS and 1× PEST (Penicillin and streptomycin antibiotics) at 37 °C and 5% CO_2_ under humidified conditions. Where appropriate, cells at 70% confluency were treated with the BRD and extraterminal (BET, BRD4) protein inhibitor thienotriazolodiazepine (JQ1; APExBIO Technology LLC, Houston, TX, USA), vitamin C, doxorubicin, 5‐fluorouracil (5‐FU), suberanilohydroxamic acid (SAHA), or the iron chelator DFO (all from Sigma‐Aldrich, St Louis, MO, USA) at different concentrations and time points following cell manipulation as per the specific experiment.

### Western Blot

Cells were washed twice with cold 1X PBS prior to lysis with radio‐immunoprecipitation assay lysis buffer containing 150 mm sodium chloride, 1.0% NP‐40, 0.1% SDS, 50 mm Tris (pH 8.0), 0.5% sodium deoxycholate, and supplemented with protease inhibitors cocktail. Cell lysates were kept on ice for 20 min and then centrifuged at 4 °C for 10 min. Supernatant was collected, and protein concentration was quantified; samples were then boiled in 5X loading buffer at 95 °C for 5 min. After separation and transfer, nitrocellulose membrane was washed with 1X TBST and blocked in 5% nonfat dry milk dissolved in 1X TBST for 1 h at room temperature. Membrane was washed three times with 1X TBST and incubated with a primary antibody against FTH (LS‐B11085‐200; LS Bio, Seattle, WA, USA; at 1 : 1000), c‐MYC (D84C12; Cell singling Technology, Danvers, MA, USA; at 1 : 1000), G9a (D5R4R; Cell Singling Technology; at 1 : 1000), or β‐actin (A 5441; Sigma‐Aldrich; at 1 : 2000) at 4 °C overnight. Primary antibody solution was discarded, and membranes were washed three times with 1X TBST and incubated with a secondary antibody [anti‐mouse (Cat. No. 7076; Cell Signaling Technology; at 1 : 1000) or anti‐rabbit antibody (ac97040; Abcam, Cambridge, UK; at 1 : 5000)] in 3% BSA in 1X TBST for 2 h at room temperature. Secondary antibody was discarded, and membrane was washed three times with 1X TBST prior to incubation with ECL solution; signal was captured using the Bio‐Rad Gel Doc system (Biorad, Hercules, CA, USA).

### Cell proliferation assay

Cell proliferation assay was performed using the MTT 3‐(4,5‐dimethylthiazol‐2‐yl)‐2,5‐diphenyltetrazolium bromide) reagent. MDA‐MB‐231, JIMT1, SKBR3, and MCF‐7 cells were separately seeded into 96‐well plates at a density of 1500 cells per well in triplicates. At 24 h postseeding, cells were manipulated and/or treated with different reagents as per the specific experiment and cultured for up to 72 h. Twenty microliter of the MTT reagent was added to each well in media and incubated for 3 h at 37 °C and 5% CO_2_ humidified conditions. Media containing MTT was then removed, and 100 μL of DMSO was added to each well to dissolve the formazan crystals. The microplate was then kept on a shaker for 5 min at room temperature to dissolve the formazan crystals in DMSO properly. Color intensity was measured/well using a 96‐well plate reader (Crocodile mini Elisa reader; BioTeck, Winooski, VT, USA) at 590 nm. Averaged optical density readings were plotted against type of treatment.

### Crystal violet staining assay

Cells were cultured in DMEM supplemented with 10% FBS and 1xPEST (penicillin and streptomycin antibiotics) at 37 °C, 5% CO_2_ under humidified conditions. After 3 days, media was removed and cells were washed with 1X PBS and fixed with colony fixation solution (one volume acetic acid/seven volumes methanol) for 15 min at room temperature. Cells were washed again carefully with 1X PBS and then stained with 0.5% crystal violet (CV) at room temperature for 20 min. CV solution was decanted, and cells were washed 3X with distilled water to remove excess dye. Colony growth was assessed visually by observing CV staining patterns in experimental vs control wells. To photograph CV‐stained cultures, plates were washed with water to remove excess dye and dried overnight, and then, images were taken using a digital camera mounted on an inverted microscope. To quantify cell proliferation using the CV assay, absorbance of CV‐stained cultures was performed. Briefly, after staining cells with 0.5% CV as described in CV staining assay section, a mixture of acetic acid and 50% ethanol (1 : 1) solution was added to each well to dissolve CV absorbed by cells. Each independent absorbance reading was performed in triplicates. Absorbance was measured at 570 nm using a 96‐well plate reader (Crocodile mini Elisa reader; BioTeck).

### Wound‐healing assay

Experimental and control cell cultures were uniformly scratched using a 10‐μL pipette tip to determine the effect of FTH1 knockdown on wound‐healing potential. The cultures were photographed, and the scratch area was measured at 24 h postscratch.

### Mammosphere formation assay

MCF‐7 cells were grown in ultra‐low attachment 96‐well plate (Corning Dow, Midland, MI, USA) as mammospheres. Mammosphere media was prepared using DMEM/F12 (Gibco, Waltham, MA, USA) supplemented with 1X B27 (Gibco), 10 ng·mL^−1^ bFGF (Invitrogen, Carlsbad, CA, USA), 20 ng·mL^−1^ EGF (Sigma‐Aldrich), and 1× PEST (penicillin and streptomycin antibiotics). Mammosphere formation cultures were maintained at 37 °C, 5% CO_2_ for 7 days before bright‐field images were taken.

### siRNA knockdown

MDA‐MB‐231, JIMT1, SKBR3, and MCF‐7 cells were seeded and allowed to grow to 50–60% confluency for 24 h prior to transfection with control siRNA and siRNA targeting FTH1 or c‐MYC or G9a using Lipofectamine RNAi‐MAX Reagent. All transfections were performed with Opti‐MEM medium. Transfection mixture was removed after 24 h, and fresh medium containing 10% FBS was added to cells for 48 h. Knockdown efficiency for both genes was confirmed by western blotting at 72 h post‐transfection. FTH1siRNA (Assay ID10829 and Catalog # AM16708), G9a siRNA (Assay ID s21468 and Catalog #4392420), and c‐Myc siRNA (Assay ID VHS40785 Catalog #1299001) were purchased from Thermo Fisher Scientific (Waltham, MA, USA).

### Transient GFP‐FTH1 overexpression

Human FT heavy chain 1/FTH1 cDNA ORF Clone C‐GFPSpark^®^ tag and control vector pCMV3‐C‐GFPSpark^®^ were purchased from Sino Biological, Beijing, China. After bacterial transformation and colony formation, both GFP‐CTL and GFP‐FTH1 plasmid DNA were purified using DNA‐spin™ Plasmid DNA Purification Kit iNtRON Biotechnology (Seoul, Gyeonggi‐do, South Korea). MDA‐MB‐231, MCF‐7, SKBR3, and JIMT1 cell lines were plated in six‐well plates and grown overnight before transient transfection. Next day, cells were transfected with GFP‐CTL and GFP‐FTH1‐encoding plasmids independently for 72 h using Lipofectamine® 3000 reagent (Thermo Fisher Scientific) according to manufacturer's instructions. A total of 2.5 μg GFP‐CTL and GFP‐FTH1 encoding plasmids were transfected independently into MDA‐MB‐231, MCF‐7, SKBR3, and JIMT1 for 72 h.

### Data mining

Gene coexpression analysis was performed on 1084 invasive BCa tissue samples, the cancer genome atlas (TCGA) dataset (Breast Invasive Carcinoma, TCGA, PanCancer Atlas, 2018) and on 921 cancer cells lines, Cancer Cell Line Encyclopedia (Broad Institute and Novartis, 2019; Broad CCLE Portal.) using the c‐BioPortal Cancer Genomics portal (http://www.cbioportal.org) [37].

### Statistical analysis

Unpaired Student's *t*‐test was used to perform statistical analysis on data related to cell growth and viability among groups; one‐way ANOVA was performed to test for statistical significance between multiple groups; *P* < 0.05 was considered as significant. Log‐rank test was used to analyze data generated using the R package from Cbioportal.

## Results

### Increased FTH1 expression inhibits cell growth and migration

**Fig. 1:**
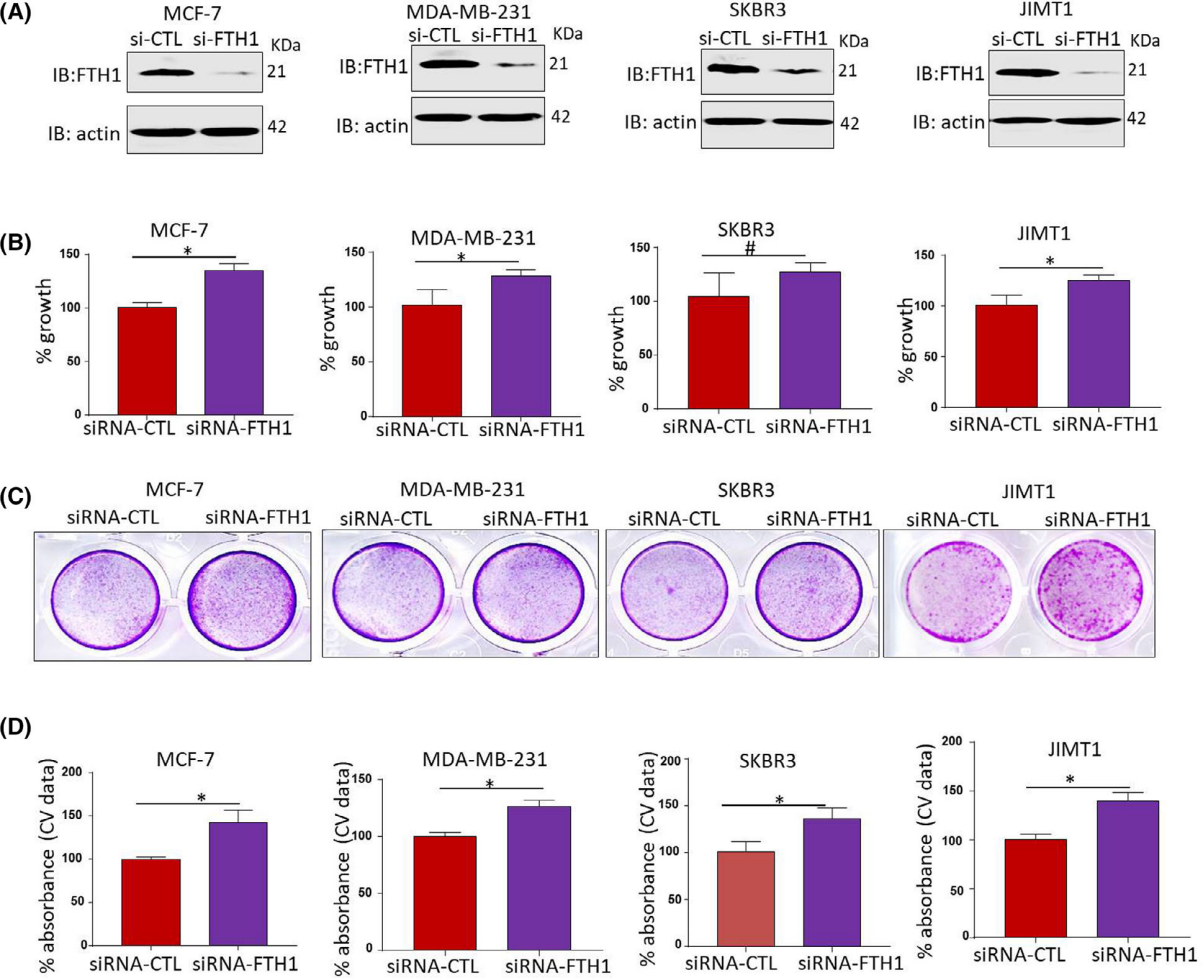
FTH1 knockdown promotes BCa cell growth. (A) Knockdown efficiency of FTH1 in MCF‐7, MDA‐MB‐231, JIMT1, and SKBR3 cell lines at 72 h post‐transfection with control or FTH1‐specific siRNA. Cell growth in MDA‐MB‐231, JIMT1, SKBR3, and MCF‐7 cells at 72 h post‐transfection with control or FTH1‐specific siRNA was evaluated by (B) MTT and (C, D) CV staining. Error bars represent the mean ± SD based on 5 (B) and 3 (D) independent experiments. Statistical analysis was performed using the Student *t*‐test; *P < 0.05; ^#^ no significant difference.

**Fig. 2:**
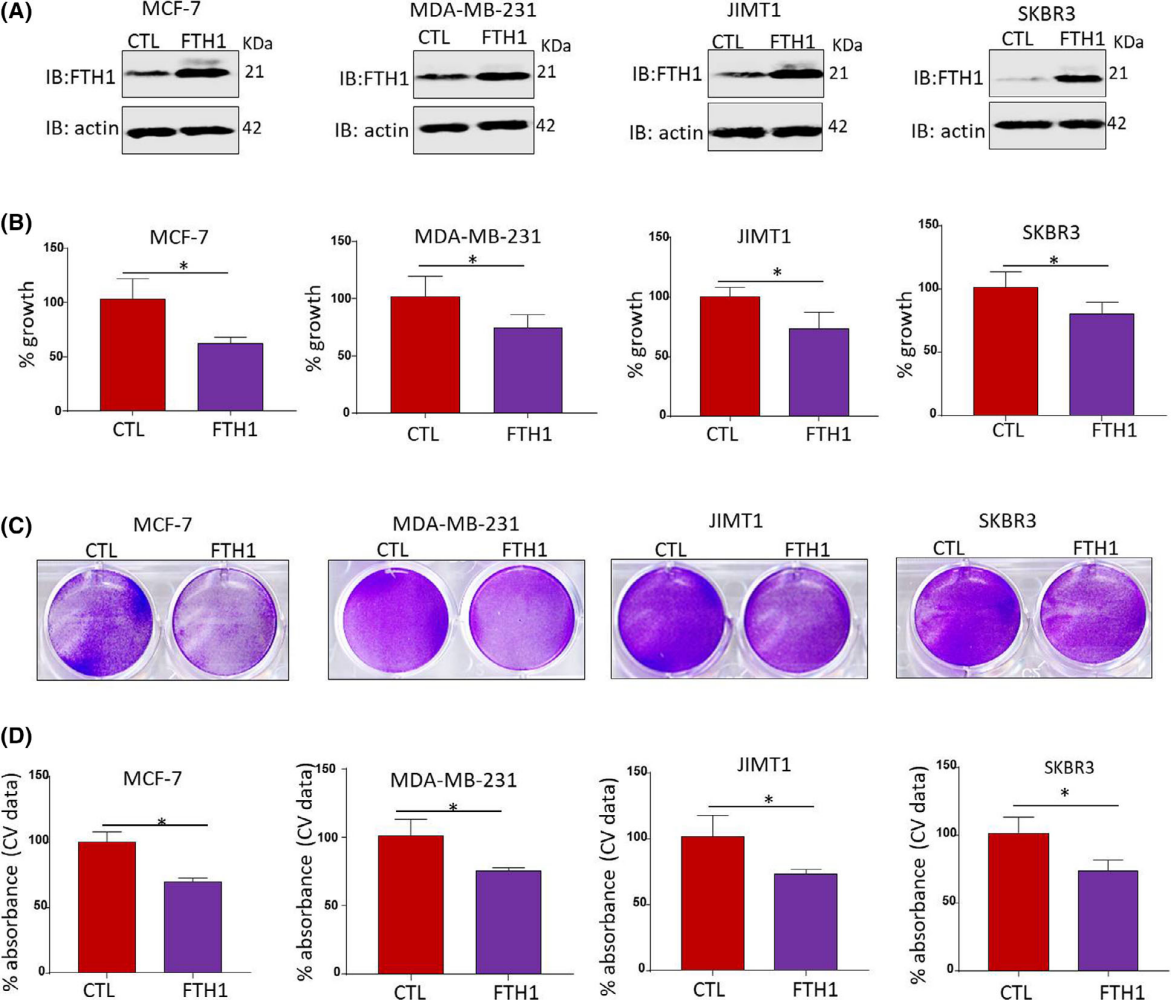
Overexpression of FTH1 inhibits BCa cell growth. (A) Overexpression efficiency of FTH1 in MCF‐7, MDA‐MB‐231, JIMT1, and SKBR3 cell lines at 72 h post‐transfection with control or FTH1‐overexpression encoding plasmids. Cell growth in MDA‐MB‐231, JIMT1, SKBR3, and MCF‐7 cells at 72 h post‐transfection with control or FTH1 overexpression was evaluated by (B) MTT and (C, D) CV staining. Error bars represent the mean ± SD based on 4 (B) and 3 (D) independent experiments. Statistical analysis was performed using the Student *t*‐test; *P < 0.05.

**Fig. 3:**
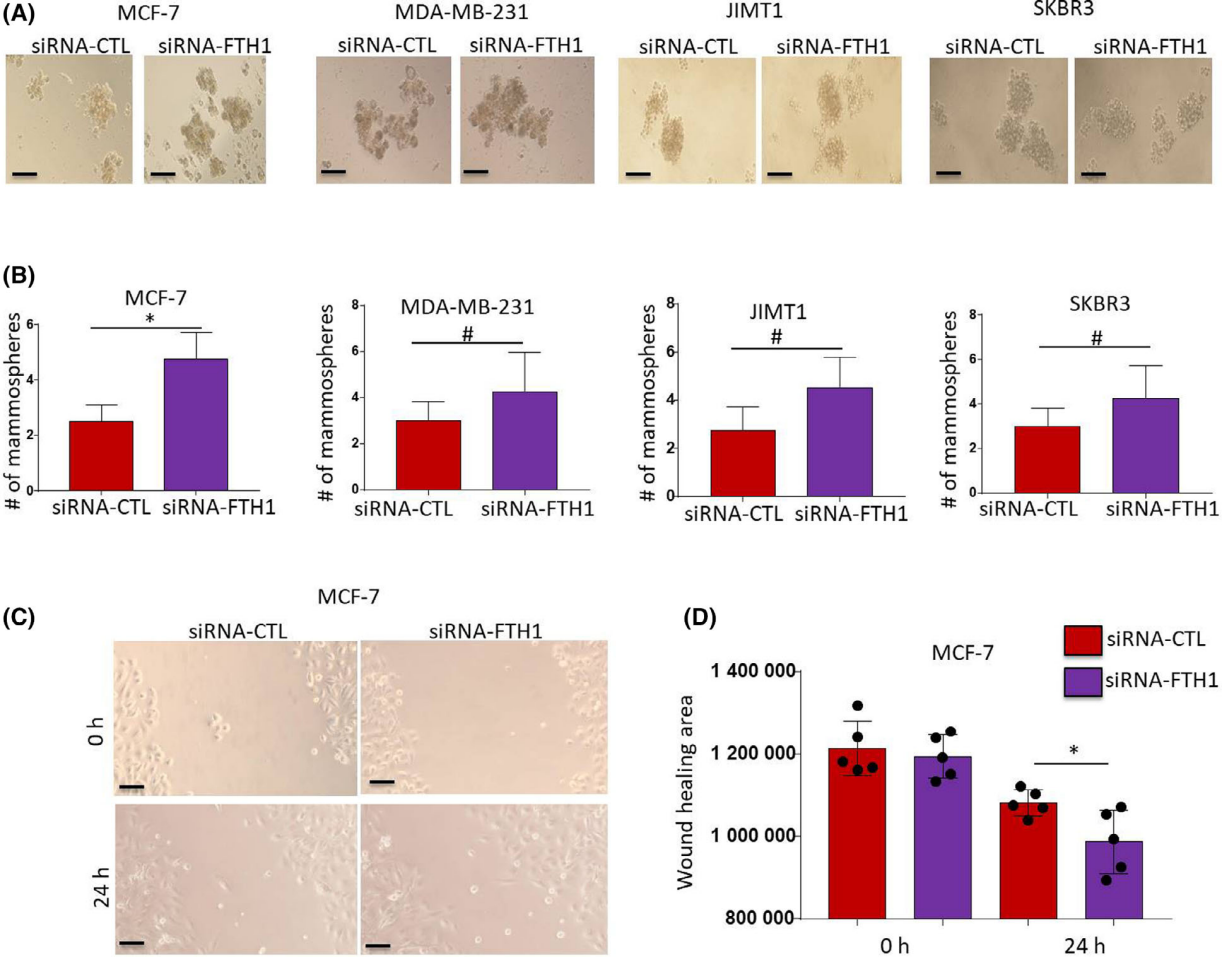
FTH1 knockdown promotes mammosphere formation efficiency and cell migration in BCa cells. (A) Mammosphere formation was evaluated in MDA‐MB‐231, JIMT1, SKBR3, and MCF‐7 cells transfected with control or FTH1‐specific siRNA and cultured in ultra‐low attachment plates for 7 days post‐transfection (20× magnification). (B) Error bars represent the mean ± SD of mammosphere numbers based on four independent experiments. (C) Wound‐healing assay was performed in MCF‐7 cells transfected with control or FTH1‐specific siRNA for 72 h post‐transfection (20× magnification); scale bar represents 200 μm. (D) Mean ± SD of wound‐healing area at 24 h postwound administration based on five independent experiments. Statistical analysis was performed using the Student *t*‐test; *P < 0.05; ^#^ no significant difference.

The effect of FTH1 knockdown on BCa cell growth was investigated in MCF‐7, MDA‐MB‐231, SKBR3, and JIMT1 BCa cells (Fig. 1A). A significant increase (*P* < 0.05) in cell growth was observed in the four cell lines at 72 h post‐FTH1 siRNA transfection as assessed by MTT (*P* < 0.05; Fig. 1B) and CV staining (Fig. 1C,D). In contrast, FTH1 overexpression (Fig. 2A) associated with a significant reduction (*P* < 0.05) in cell growth in the four cell lines; growth reduction in MCF‐7 cells transfected with GFP‐FTH1 vector was particularly significant (*P* = 0.0067; Fig. 2B). CV staining also showed inhibition of cell growth in BCa cells transfected with GFP‐FTH1 vector (Fig. 2C,D). We next assessed the effect of FTH1 knockdown on mammosphere formation. Higher mammosphere numbers were observed in FTH1‐silenced cells relative to siRNA controls; this was especially true for MCF‐7 cells (Fig. 3A,B). The effect of FTH1 silencing on cell migration was also indirectly examined using the wound‐healing assay. As shown in (Fig. 3C,D), greater cell migration occurred in cultures of FTH1‐silenced MCF‐7 cells relative to siRNA controls.

### FTH1 silencing increases c‐MYC and G9a expression and enhances cell growth

**Fig. 4:**
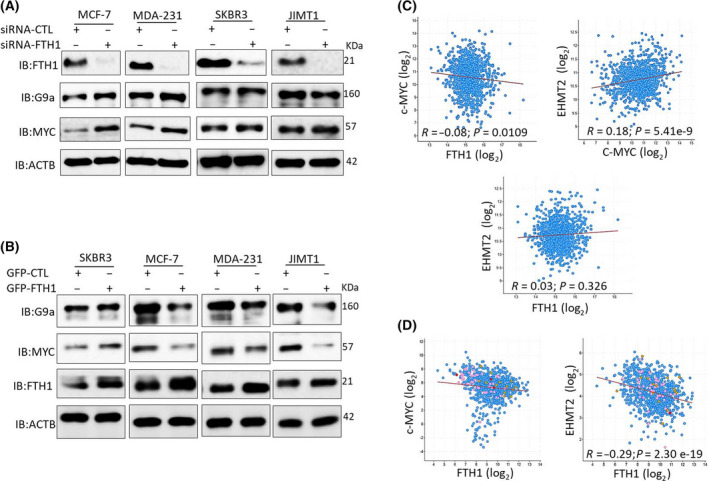
FTH1 expression negatively correlates with c‐MYC and G9a in BCa cell lines and tissues. (A) Expression of FTH1, c‐MYC, and G9a was evaluated by western blotting in MDA‐MB‐231, JIMT1, SKBR3, and MCF‐7 cells at 72 h post‐FTH1 silencing. (B) Expression of FTH1, c‐MYC, and G9a was evaluated by western blotting in MDA‐MB‐231, JIMT1, SKBR3, and MCF‐7 cells at 72 h post‐FTH1 overexpression. Coexpression analysis of FTH1 vs c‐MYC, FTH1 vs G9a (EHMT2), and c‐MYC vs G9a in (C) 1084 BCa tissue samples (Breast Invasive Carcinoma, TCGA, PanCancer Atlas, 2018) and (D) in 921 cancer cell lines (Broad Institute and Novartis, 2019; Broad CCLE Portal.) using the c‐BioPortal Cancer Genomics portal.

**Fig. 5:**
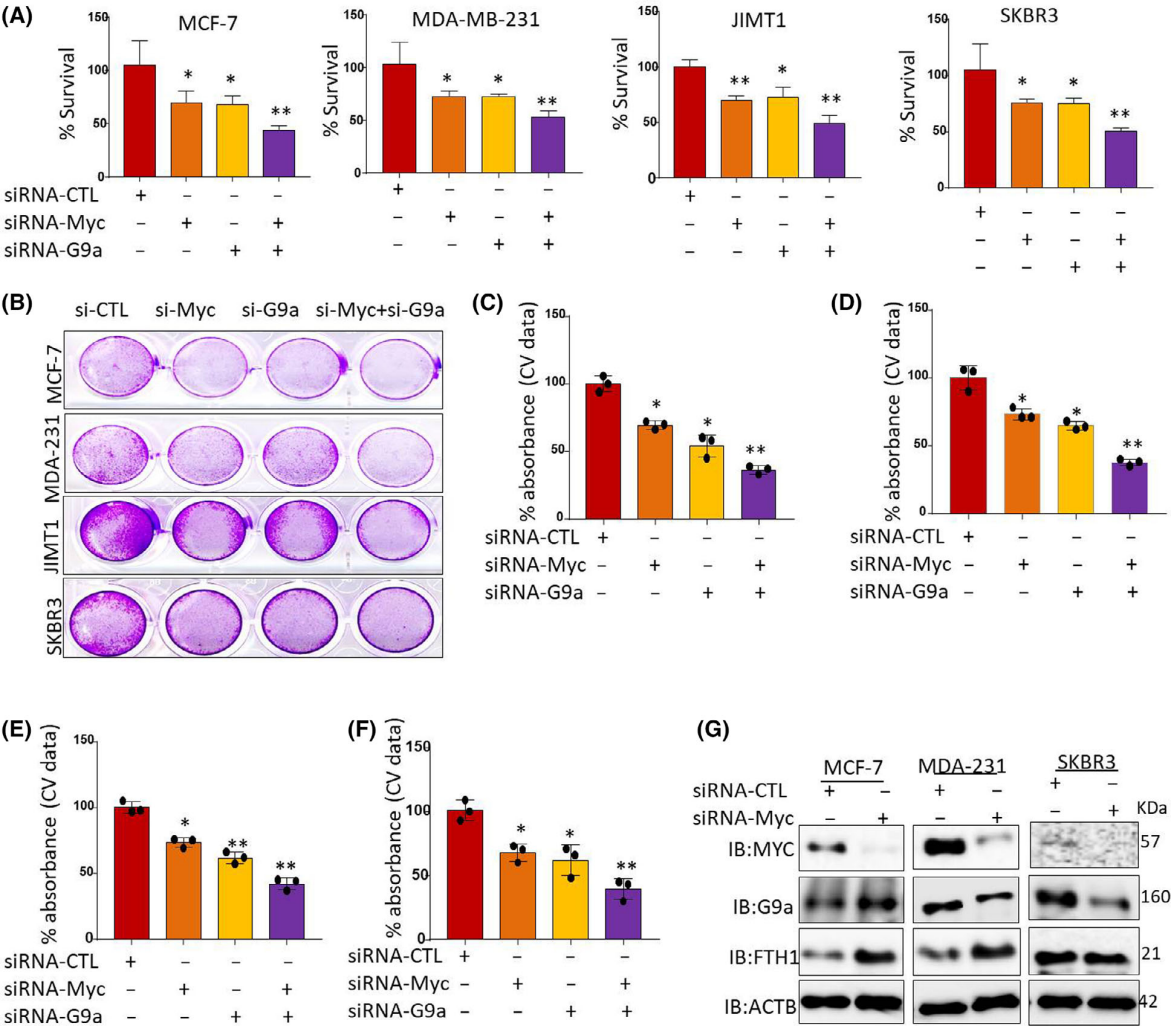
Silencing of c‐Myc and/or G9a upregulates FTH1 and inhibits BCa cell growth. Cell growth was assessed by (A) MTT and (B) CV staining in c‐MYC‐ and/or G9a‐silenced MDA‐MB‐231, MCF‐7, SKBR3, and JIMT1 cells at 72 h post‐transfection; error bars in A represent the mean ± SD based on four independent experiments. (C–F) Mean ± SD of CV absorbance values based on three independent experiments. *t*‐Test was performed. *P < 0.05; **P < 0.005. (G) Expression of FTH1, c‐MYC, and G9a in c‐MYC‐silenced MDA‐MB‐231, SKBR3, and MCF‐7 cells at 72 h post‐transfection; cells transfected with control siRNA served as negative control; β‐actin was used as a loading control.

Ferritin heavy chain was previously shown to inhibit the expression of several oncogenes including CXCR4/CXCL12, mir‐638, miR‐125b, CCND1, p‐AKT, and BCL2 [38, 39, 40]. However, its effect on the expression of key oncogenes involved in cellular iron metabolism such as c‐MYC [41] and G9a [42] remains largely unknown. Moreover, assessing the effects of FTH1 on c‐MYC expression was of particular interest given that the latter is known to downregulate FTH1 expression in cancer cells [41]. To this end, the expression of c‐MYC and G9a was assessed in FTH1‐silenced MCF‐7, MDA‐MB‐231, JIMT1, and SKBR3 cells. As shown in Fig. 4A, FTH1 silencing resulted in increased c‐MYC expression in the four cell lines. G9a expression also increased in FTH1‐silenced MCF‐7, MDA‐MB‐231, and SKBR3 cells but not JIMT1 cells. The effect of FTH1 overexpression on c‐MYC and G9a expression was also examined in MCF‐7, MDA‐MB‐231, JIMT1, and SKBR3 cells transfected with the GFP‐FTH1 vector. MCF‐7, MDA‐MB‐231, and JIMT1 cells overexpressing FTH1 showed strong inhibition of c‐MYC and G9a expression (Fig. 4B). In contrast, SKBR3 cells overexpressing FTH1 showed increased c‐MYC and G9a expression relative to GFP‐CTL controls. The inverse correlation between FTH1 and c‐MYC or G9a was examined in BCa tissue samples using the TCGA database gene expression analysis tools. As shown in Fig. 4C, FTH1 expression negatively correlated with that of c‐MYC; no clear correlation was evident between FTH1 and G9a. Additionally, a clear positive correlation was observed between c‐MYC and G9a. A similar pattern of correlation between FTH1 and c‐MYC or G9a was observed in 921 cell lines representing different types of human cancer (Fig. 4D). Silencing of c‐MYC or G9a resulted in reduced growth in MCF‐7, MDA‐MB‐231, JIMT1, and SKBR3 cells as assessed by MTT (Fig. 5A) and CV (Fig. 5B–F). Interestingly, cosilencing of both genes resulted in a significant reduction in cell growth relative to controls (> 60%) or to c‐MYC‐ or G9a‐silenced cells (> 30%) as assessed by MTT (Fig. 5A) and CV (Fig. 5B–F) in all cells tested. Silencing of c‐MYC also associated with increased FTH1 expression in MCF‐7, MDA‐MB‐231 but not in SKBR3 cells; it also resulted in reduced G9a expression in MDA‐MB‐231 and SKBR3 cells (Fig. 5G).

### Increased c‐MYC expression correlates with increased iron influx and reduced iron storage and release in cancer cells

**Fig. 6:**
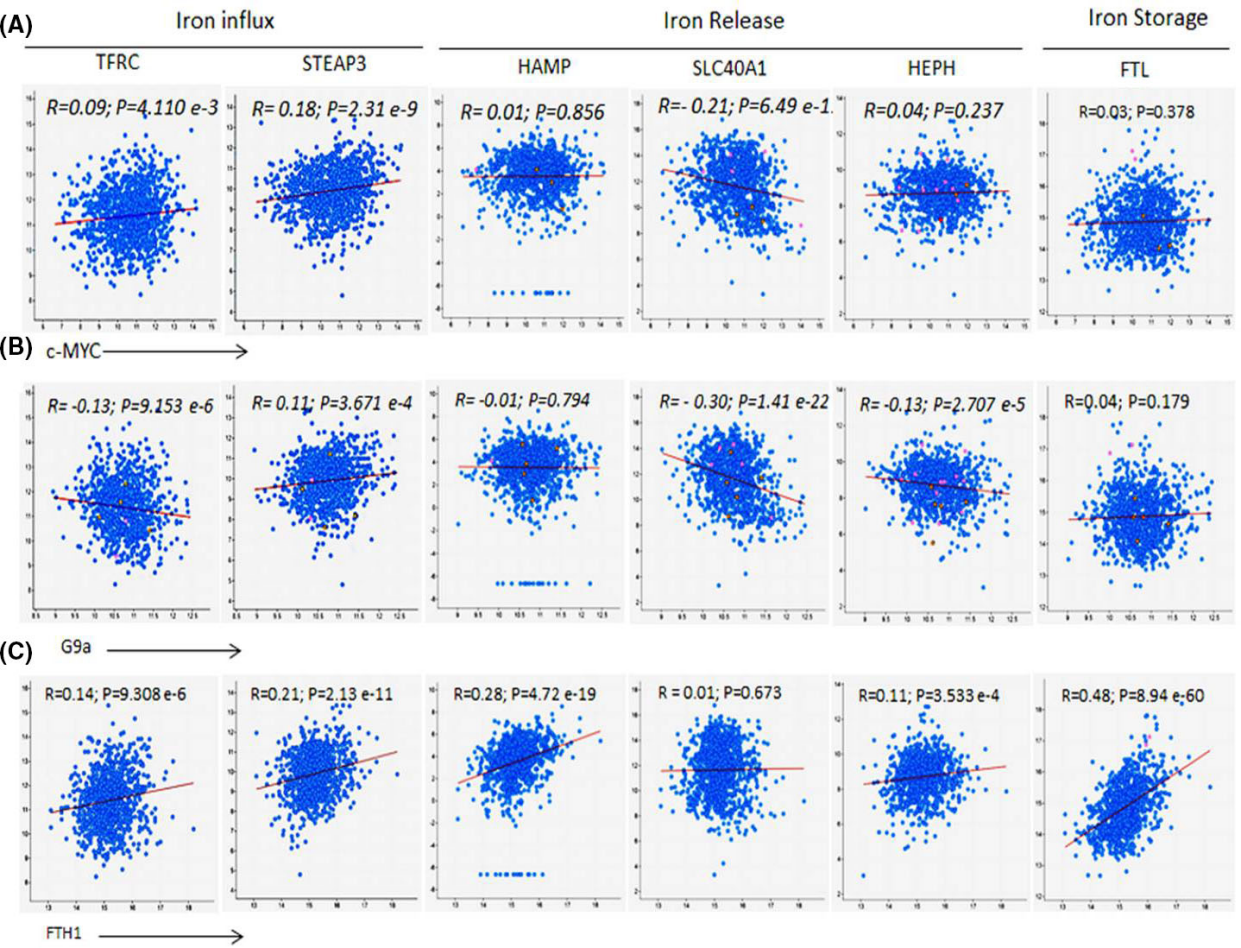
Correlation of FTH1, MYC, and G9a with key IRGs in BCa samples. mRNA coexpression analysis of (A) c‐MYC, (B) G9a, or (C) FTH1 vs genes that encode the TfR1 gene (TFRC), STEAP3, hepcidin (HAMP), FPN (SLC40A1), hephaestin (HEPH), and FTL chain using TCGA BCa dataset (1084 patient samples; http://www.cbioportal.org); *R* and *P* values were generated using Pearson statistic.

Previous work has shown that c‐MYC increases labile cellular iron availability by repressing FTH1 expression and reducing iron‐storing potential in cancer cells [41]. Additionally, G9a was previously reported to limit cellular iron release potential in BCa cell by repressing ferroxidase hephaestin expression [42]. These observations raised the question of whether repressing c‐MYC and G9a expression by FTH1 may further modulate cellular iron metabolism in cancer cells in such a way that reduces cell growth potential. To test this hypothesis, coexpression analysis involving FTH1, c‐MYC, or G9a vs iron regulatory genes (IRGs) encoding including TfR1 (TFRC), the metalloreductase six‐transmembrane epithelial antigen of the prostate 3 (STEAP3), hepcidin (HAMP), FPN (SLC40A1), hephaestin (HEPH), and FT light chain (FTL) was carried out on BCa samples using TCGA datasets. As shown in Fig. 6, c‐MYC expression correlated positively with that of TFRC and STEAP3 and negatively with that of SLC40A1; no clear correlation was evident between c‐MYC and HAMP, HEPH or FTL. G9a expression correlated positively with that of STEAP3 and negatively with that of HAMP, HEPH, and SLC40A1; no correlation with HAMP or FTL was evident. Surprisingly, G9a expression correlated negatively with that of TFRC. FTH1 expression correlated positively with that of TFRC, STEAP3, HAMP, HEPH and FTL1; no clear correlation was evident between FTH1 and SCL40A1 (Fig. 6).

### Reduced c‐MYC and G9a expression enhances BCa cell sensitivity to chemotherapy

**Fig. 7:**
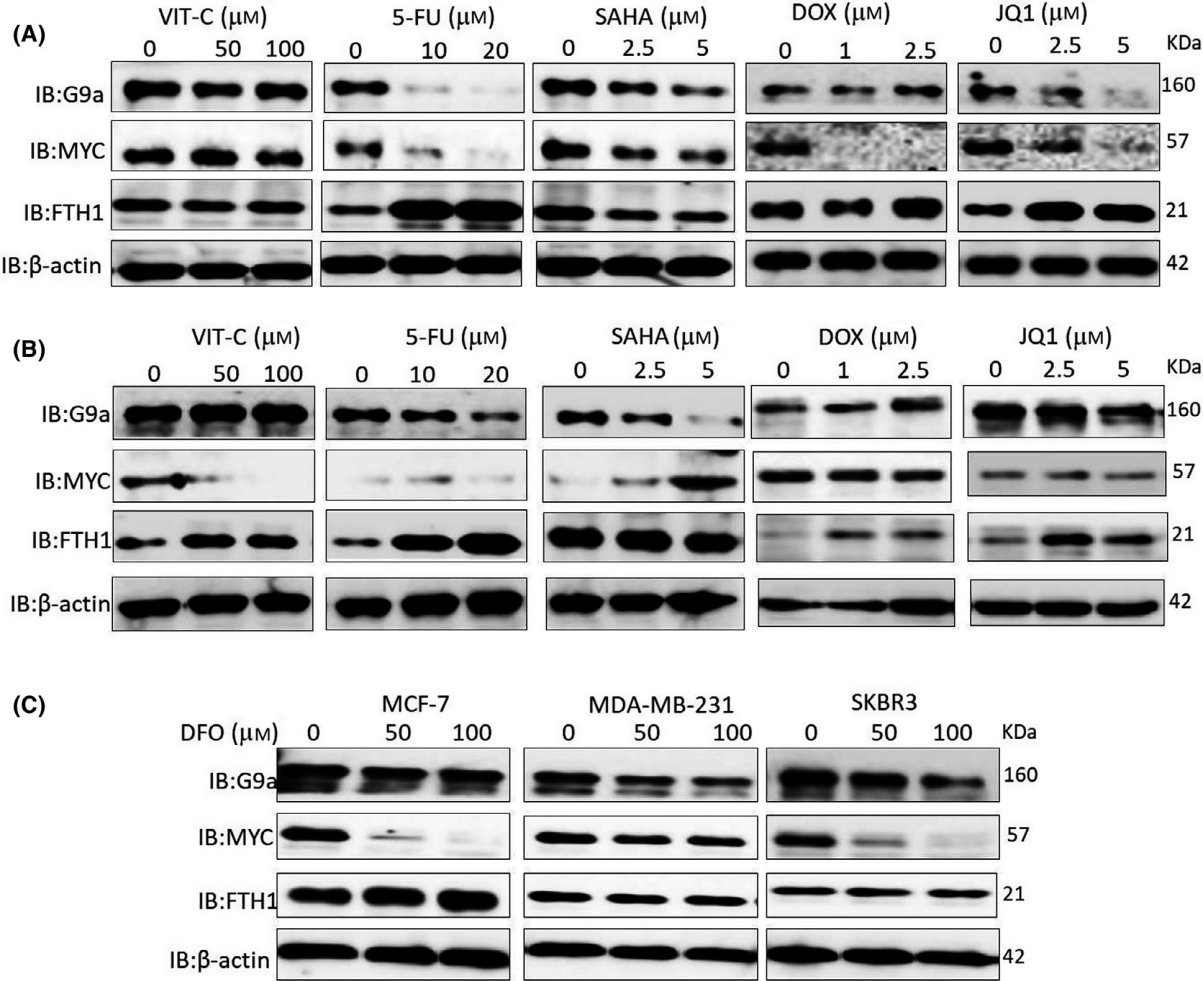
Anticancer drugs and iron chelation reduce c‐MYC and G9a expression and increase FTH1 expression in BCa cells. (A) MCF‐7 and (B) MDA‐MB‐231 cells treated with the indicated doses of vitamin C, 5‐FU, SAHA, doxorubicin, and JQ1 for 48 h were assessed for the expression of FTH1, c‐MYC, and G9a by western blot. (C) The expression of FTH1, c‐MYC, and G9a was also examined in MCF‐7, MDA‐MB‐231, and SKBR3 cells treated with 50 or 100 µm of DFO for 48 h relative to untreated controls. β‐Actin was used as a loading control.

Given that FTH1 negatively impacts the expression of c‐MYC and G9a along with cell growth and migration, the expression status of these three genes was examined at the protein level in cells separately treated with the BET (BRD4) protein inhibitor thienotriazolodiazepine (JQ1), vitamin C, the anticancer drugs doxorubicin and 5‐FU, the epigenetic modifier SAHA, and the iron chelator DFO as means of assessing its prognostic value in chemotherapy. Treatment with 5‐FU, doxorubicin, and JQ1 showed a significant reduction in c‐MYC and G9a expression and a significant increase in FTH1 expression in MCF‐7 and MDA‐MB‐231 cells (Fig. 7A). Reduced expression of all three proteins was observed in SAHA‐treated MCF‐7 cells. G9a expression was inhibited in MDA‐MB‐231 cells treated with 5‐FU, SAHA, and JQ1 in MDA‐MB‐231 (Fig. 7B). Treatment of MDA‐MB‐231 cells with vitamin C, 5‐FU, doxorubicin, or JQ1 also resulted in a significant increase in FTH1 expression; SAHA did not change FTH1 expression status in these cells. Iron chelation (DFO) also resulted in reduced c‐MYC and G9a expression in MDA‐MB‐231, MCF‐7, and SKBR3 cells (Fig. 7C). However, only MCF‐7 cells showed increased FTH1 expression in response to DFO treatment.

**Fig. 8:**
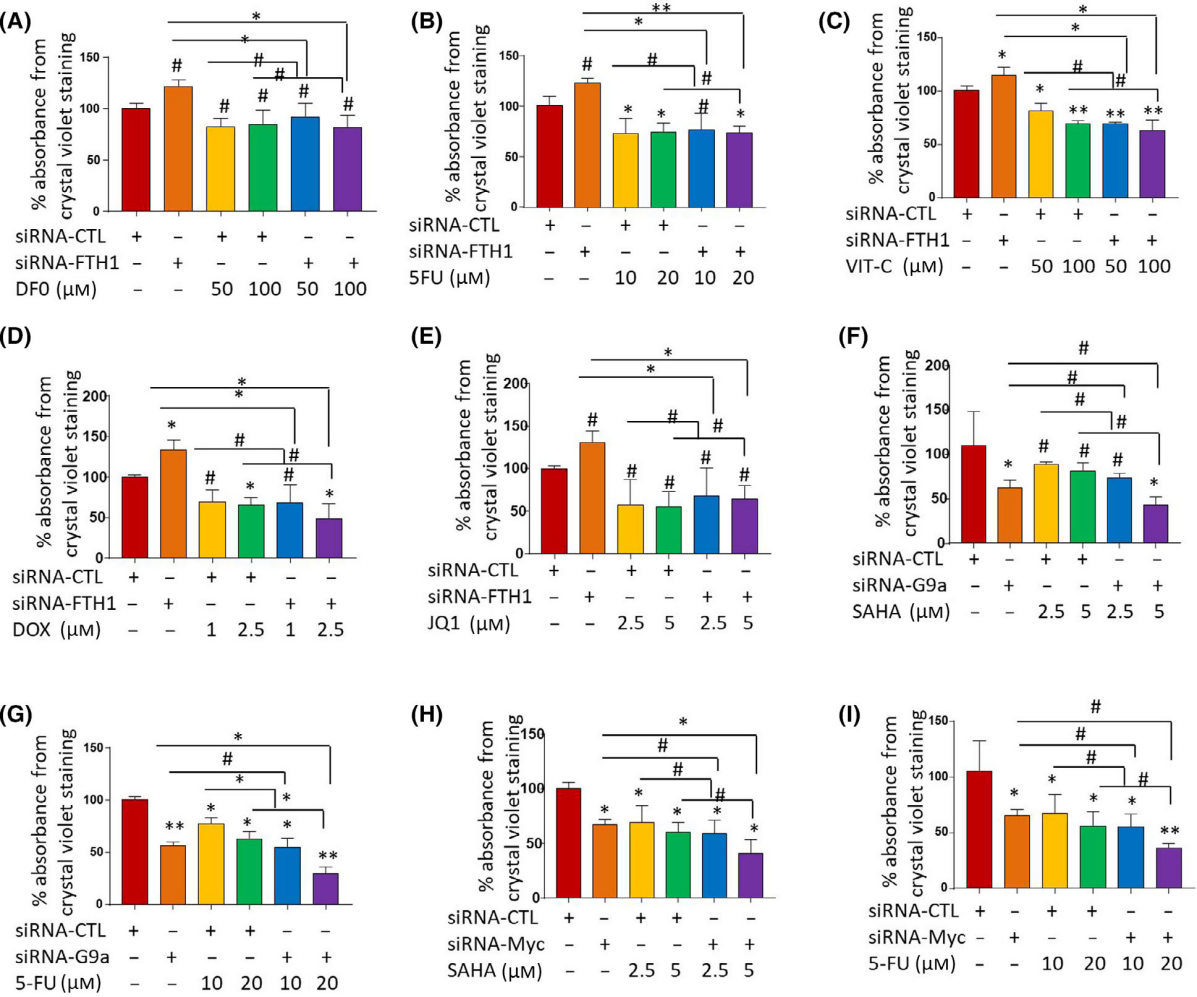
Reduced c‐MYC and G9a expression associates with enhanced cell sensitivity to chemotherapy. Viability of MCF‐7 cells silenced for FTH1 24 h prior to treatment with increasing concentrations of (A) DFO, (B) 5‐FU, (C) vitamin C, (D) doxorubicin, or (E) JQ1. (A‐E) Error bars show the mean ± SD from four replicated experiments. Cell viability of MCF‐7 cells silenced for G9a 24 h prior to treatment with increasing concentrations of (F) SAHA or (G) 5‐FU or silenced for c‐MYC 24 h prior to treatment with increasing concentrations of (H) SAHA or (I) 5‐FU. MCF‐7 cells silenced for c‐MYC 24 h prior to treatment with increasing concentrations of SAHA or 5‐FU. Cell viability was assessed by CV staining at 72 h post‐treatment. (F, I) Error bars show the mean ± SD from four replicated experiments. One‐way ANOVA was performed to test for statistical significance among multiple groups; *P < 0.05, **P < 0.005, ^#^ not significant.

The observation that FTH1 expression increases in response to chemotherapy raised the question of whether inhibition of FTH1 can render tumor cells more resistant to chemotherapy. To address this question, cell growth was examined in FTH1‐silenced MCF‐7 cells treated with increasing concentrations of DFO, vitamin C, 5‐FU, JQ1, and doxorubicin. FTH1 knockdown promoted cell growth while DFO, vitamin C, 5‐FU, JQ1, and doxorubicin treatment reduced it (Fig. 8A–E). Moreover, a minimal increase in cell growth was observed in FTH1‐silenced cells following treatment with any of these agents. Given that 5‐FU and SAHA can inhibit c‐MYC and G9a expression (Fig. 7) and that upregulated expression of c‐MYC and/or G9a increases resistance to chemotherapy, the question of whether inhibition of c‐MYC and G9a expression prior to treatment with 5‐FU or SAHA can modulate the response to these agents was also addressed in c‐MYC‐ or G9a‐silenced MCF‐7 cells and subsequently treated with increasing concentrations of 5‐FU and SAHA. Knockdown of G9a (Fig. 8F,G) or c‐MYC (Fig. 8H,I) resulted in a significant dose‐dependent increase in MCF‐7 sensitivity to SAHA and 5‐FU.

## Discussion

Despite major advances, BCa treatment remains a clinical challenge. A deeper understanding of cellular iron metabolism in BCa cells may prove beneficial in both prognosis and therapy. Data presented here clearly show that FTH1 is a significant tumor suppressor in BCa cells. In that, FTH1 silencing promoted BCa cell growth and migration and associated with increased c‐MYC and G9a expression. In contrast, FTH1 overexpression inhibited BCa cell growth and migration and associated with decreased c‐MYC and G9a expression. This is consistent with previously published work which has demonstrated that reduced FTH1 expression associates with increased tumor growth and progression [16, 23] and that reduced FTH1 [41] or FT as a whole [43] is prerequisites for cell transformation. Previous work has also demonstrated that FTH1 can directly interact with and activate p53 and that its expression tends to be at a much lower level in p53‐mutant BCa types relative to wild‐type counterparts [23, 27]. FTH1 silencing was previously reported to severely impair ERK1/2 phosphorylation and to significantly reduce RAF1 expression in a miR‐125b‐5p‐dependent manner [44]. The inference that FTH1 is a tumor suppressor in BCa is also consistent with the observation that reduced c‐MYC and/or G9a increased the sensitivity of BCa cells to anticancer drugs [45, 46, 47]. Although BCa tissues tend to be FT‐rich [13, 47, 48], the FTL/FTH1 ratio in BCa can reach up to 6/1, suggesting that increased FT expression in BCa is due to FTL rather than FTH1 overrepresentation [49].

Similar to the effects of FTH1 overexpression, c‐MYC and/or G9a silencing associated with increased FTH1 expression and inhibited BCa cell growth. This is consistent with previous work which has shown that FTH1 expression is negatively regulated by key oncoproteins such as c‐MYC [22] and E1A [50] and is positively regulated by p53 [23] and proinflammatory cytokines TNF‐α and IL‐1α [51]. Coexpression analysis also showed c‐MYC or G9a expression to be negatively correlated with that of FTH1 in BCa tissue samples. It also showed that overall, c‐MYC and G9a tended to correlate positively with IRGs that enhance iron influx (TFRC and/or STEAP3) and negatively with IRGs that enhance cellular iron release (HAMP and SCL40A1); no significant correlation was observed between c‐MYC and HAMP or the ferroxidase HEPH. Taken together, these observations suggest that increased expression of c‐MYC and/or G9a helps cancer cells to increase cellular iron availability by increasing iron influx while at the same time reducing its storage and release. This tentative assumption is in line with previous work which has shown that c‐MYC stimulates the expression of IRP‐2, which increases cellular iron availability [41]. It is also in agreement with the observation that G9a represses the ferroxidase hephaestin expression as means of limiting cellular iron release [32]. Interestingly, our study demonstrated that iron chelation associates with increased FTH1 expression and decreased c‐MYC and G9a expression. This lends further support to the possibility that cellular iron content in BCa cells could be a major target for the opposing modulatory effects of oncogenes such as c‐MYC and G9a vs tumor suppressor genes such as FTH1 [17, 19, 22, 23, 28]. This is further supported by previous work which has demonstrated that iron chelation in MCF‐7 and MDA‐MB‐231 cells resulted in a significant decrease in intracellular labile iron, which associated with increased expression of hepcidin, FT, and TfR1 [52]. That said, other chelation‐related factors such as type and dose of chelator, exposure time, target cell type, and the mode of chelator internalization by target cells may differentially influence the pattern of disruption in cellular iron metabolism.

## Conclusions

This study has identified a novel feedback loop that governs the relationship between FTH1 and the oncogenes c‐MYC and G9a in BCa cells. Through this novel interplay, decreased FTH1 expression increases the expression of c‐MYC and G9a and enhances BCa cell growth. In contrast, decreased c‐MYC and/or G9a expression increases the expression of FTH1 and G9a and reduces BCa cell growth. More work is still needed to understand how this negative feedback induction loop operates and whether it involves direct interaction between FTH1 and c‐MYC or G9a. These observations suggest that FTH1 is a tumor suppressor in BCa cells and that increased FTH1 expression may signify a favorable prognosis and response to chemotherapy.

## Conflict of interest

The authors declare no conflict interest.

## Author contributions

MaH and AA were responsible for the conception of the idea, data analysis, and manuscript preparation; AA, JS, RAJ, JSM, NA, MoH, and HU performed the experimental work; MaH, AA, MoH, and JSM analyzed the data; MaH, JSM, and AA drafted and edited the manuscript.

## Data Availability

All pertinent data are included in the manuscript.
